# Developing image analysis methods for digital pathology

**DOI:** 10.1002/path.5921

**Published:** 2022-05-23

**Authors:** Peter Bankhead

**Affiliations:** ^1^ Edinburgh Pathology, Institute of Genetics and Cancer University of Edinburgh Edinburgh UK; ^2^ Centre for Genomic & Experimental Medicine, Institute of Genetics and Cancer University of Edinburgh Edinburgh UK; ^3^ Cancer Research UK Edinburgh Centre, Institute of Genetics and Cancer University of Edinburgh Edinburgh UK

**Keywords:** digital pathology, computational pathology, image processing, image analysis, open science, software

## Abstract

The potential to use quantitative image analysis and artificial intelligence is one of the driving forces behind digital pathology. However, despite novel image analysis methods for pathology being described across many publications, few become widely adopted and many are not applied in more than a single study. The explanation is often straightforward: software implementing the method is simply not available, or is too complex, incomplete, or dataset‐dependent for others to use. The result is a disconnect between what seems already possible in digital pathology based upon the literature, and what actually is possible for anyone wishing to apply it using currently available software. This review begins by introducing the main approaches and techniques involved in analysing pathology images. I then examine the practical challenges inherent in taking algorithms beyond proof‐of‐concept, from both a user and developer perspective. I describe the need for a collaborative and multidisciplinary approach to developing and validating meaningful new algorithms, and argue that openness, implementation, and usability deserve more attention among digital pathology researchers. The review ends with a discussion about how digital pathology could benefit from interacting with and learning from the wider bioimage analysis community, particularly with regard to sharing data, software, and ideas. © 2022 The Author. *The Journal of Pathology* published by John Wiley & Sons Ltd on behalf of The Pathological Society of Great Britain and Ireland.

## Introduction

The growth of digital pathology and whole‐slide imaging have created the opportunity to extract more information from histological samples through image analysis. However, despite considerable progress over the last decade, digital pathology analysis remains difficult to employ in practice and much of its promise remains to be fulfilled.

The core ideas of image analysis are quite straightforward, although applying them is not. A digital image is composed of pixels. In pathology, most images are brightfield whole‐slide scans in RGB format: this means that each pixel comprises three numbers—usually 8‐bit integers in the range 0–255—that together represent the red, green, and blue components of the colour used to display the pixel. A typical whole‐slide image can therefore be thought of as a *Width × Height × 3* array; the width and height often exceed 100,000 pixels each, so the raw data comprises billions of numbers. The challenge of analysis is to identify and interpret meaningful patterns within these numbers—and to do so in a way that is robust to variation from multifarious sources, including biology, tissue processing, staining, and scanning. Even a small study containing tens of images requires us to grapple with trillions of pixels, from which we often want to extract at most a few actionable insights per image.

The richness of histopathology imaging data means that a single whole‐slide scan affords a plethora of possibilities for analysis. Ideally, our choice would be driven by the precise question we want to answer from the image. In practice, we are limited by the tools at our disposal and how we use them. Most pathologists do not write software, while few algorithm and software developers know very much about pathology; if digital pathology software is to be useful, therefore, both sides need to be able to communicate effectively with one another. This involves having an accurate perception of the strengths and limitations of each discipline, focussing on areas where computational methods can have a meaningful impact.

Numerous recent digital pathology and artificial intelligence (AI) reviews provide an excellent overview of progress towards clinical applications [1, 2, 3, 4, 5, 6]. Here, I aim to offer a practical assessment of the use and development of digital pathology tools in research today. After distinguishing between approaches and techniques, I describe some of the main challenges faced by people who currently use digital pathology tools to analyse their data. This review ends with a discussion concerning how digital pathology could benefit from insights and practices from the broader field of bioimage analysis, particularly with regard to open data and software.

## Approaches to digital pathology analysis

Digital pathology analysis can be broadly categorised into two main approaches: quantitative analysis and AI‐driven assessment. Although any specific application might require a combination of both, one approach usually dominates. Making this distinction can help to elucidate issues concerning how algorithms are developed and validated, and where a pathologist's knowledge fits into the digital system.

### Quantitative analysis

Historically, pathology image analysis has focussed on some combination of detecting, classifying, counting, and/or quantifying specific ‘objects’ visible within the image. These objects may be of different kinds and identified at different scales. For example, determining whether a slide contains evidence of invasive primary tumour [7], or metastatic tumour [8], or *Helicobacter pylori* infection [9] are all primarily detection tasks. Often, what is detected needs to also be classified; for example, nuclei might be classified according to different cell types, and this used to determine metrics predictive of therapeutic response, such as the relative proportions of lymphocytes and tumour cells [10]. Alternatively, we might eschew cell detection in favour of classifying pixels directly as belonging to tumour epithelium, stroma, or other tissue types, and from this quantify the areas occupied by each tissue class (for example, to assess fat proportion [11], or the tumour‐stroma ratio [12, 13]). Each of the suggested analyses could be applied to the same whole‐slide scan of a tissue section, depending upon the questions we wish to address.

The common feature of such quantitative analysis is that it is concerned with assessing something that is well‐defined and visible. *In principle*, it replicates what a pathologist could ascertain by looking at a slide. A knowledgeable observer can determine whether the analysis has been performed appropriately by visualising what has been detected, classified, and quantified.

### AI‐based assessment

The problems that may be addressed through quantitative analysis are often surrogates for what we really want to know. This includes questions concerning what diagnostic or prognostic information we can determine from the image, or to which treatment a specific patient is likely to respond.

Much recent work in digital pathology has focussed on demonstrating how such questions may be approached more directly—*without* explicitly detecting or quantifying specific features. An early example demonstrated that AI techniques could predict mutations in six commonly mutated genes in lung adenocarcinoma [14]. Similar strategies have since been applied to predict mutations for many more genes across a wide range of tissue types [15, 16, 17, 18, 19]. Other studies have shown that AI may be used to directly predict patient outcome from hematoxylin and eosin [H&E] slide scans [20, 21, 22, 23].

In these cases, the output is a prediction that is not based on directly measuring any particular feature or structure. This makes verifying the result more difficult, at least on a per‐image basis. Confidence needs to be earned through: (1) large‐scale validation studies using diverse datasets, and (2) the ability to visualise regions of the slide that contributed more or less strongly to the result. Such visualisations can reveal that the AI has learned to base its predictions of regions of the image that are already known to be clinically relevant [15, 16].

### Hybrid approaches

While not all digital pathology applications fit neatly into the two categories above, we may still distinguish between outputs that are amenable to visual verification and those that are not. For example, numerous AI‐based methods have been developed for Gleason grading [24, 25]. Some include elements of object detection and quantification before the application of AI, whereas others operate more directly on the pixels without explicit detection; either way, a pathologist can judge the algorithm's performance by comparing the final generated gradings with their own assessments. This differs from an AI‐based approach to prostate cancer risk stratification [26] or cancer recurrence prediction [27], designed without recourse to any established grading system, which is less amenable to visual verification—and therefore perhaps less appealing to pathologists in terms of adoption.

## Techniques for image analysis

Regardless of approach, the fundamental challenge of digital pathology remains the same: to uncover patterns among the pixels. This involves applying mathematical operations to the numbers in the input image, typically in a way that progressively transforms the image into a form in which the key features can be separated from everything else. While each individual operation may be straightforward, complexity ensues whenever hundreds—or even thousands—of such operations are combined into an algorithm that is applied to billions of pixels. Nevertheless, recognising the essential simplicity of building blocks used to construct digital pathology algorithms is central to understanding their strengths and predicting their limitations. In some cases, the success or failure of a complex analysis can come down to a single cutoff threshold applied at a key step.

### Conventional image processing

Conventional image processing involves explicitly defining the processing operations involved, typically drawn from a wide range of established techniques. For example, processing an H&E image often begins with stain separation by *colour deconvolution* [28]; this effectively recombines the red, green, and blue values for each pixel using a weighted sum characterised by the stain colour. This may be followed by a *convolution*: an image filtering operation that replaces each pixel by a weighted sum of the neighbouring pixels; the weights are defined by a *convolution kernel*, and different kernels result in output images that highlight different kinds of feature at different scales (e.g. filamental structures, edges, blobs of different sizes). Convolution is often applied to duplicates of the image using different kernels, then recombined by adding or subtracting corresponding pixels. Eventually, an image is produced wherein the pixel values corresponding to structures of interest can be separated from all other pixels by applying a *threshold*, thereby generating a binary image representing distinct objects that can be measured. Some additional operations may be needed (e.g. nonlinear filters, distance or watershed transforms) to adequately split clustered objects or refine boundaries. A link to an overview of these techniques is provided in the ‘Data availability statement’ section.

The digital pathology literature is replete with image processing publications at all levels: describing individual operations (e.g. new approaches to stain separation), combinations of operations for generic tasks (e.g. nucleus detection), and full algorithms devised for specific applications (e.g. Ki67 evaluation in breast cancer). In each case, the processing is hand‐crafted and deterministic. Core operations can be endlessly adjusted and recombined to construct different algorithms for different purposes.

### Machine learning

Crafting robust image processing algorithms requires substantial effort and a good understanding of the data. It also takes imagination: the developer needs to guard against the many ways in which the algorithm might fail on unseen images, artefacts, and anomalies. In practice, this can never be entirely successful: it is hard to think of everything that might go wrong, and even recognised problems are hard to overcome.

This would be easier if the computer could exhibit human‐like intelligence, informed by example and experience. Such AI can be achieved (to an extent) by using the techniques of machine learning to train a model capable of making useful predictions on new data. Here, we will focus on supervised machine learning, whereby the model is trained to make predictions using labelled data with a defined target. This is in contrast to unsupervised approaches, which may be used to find clusters in data whenever labels are unavailable [29].

Developing a supervised machine‐learning algorithm for digital pathology requires inputs with associated labels, a model capable of making predictions from the inputs, and a loss function that computes an error between a prediction and a label. The goal during training is to iteratively refine the model until the loss between predictions and labels is minimised. The concept is very generic, and labels take different forms depending upon the task at hand. For example, regions annotated by a pathologist might be used to derive a label for every pixel of an image, and used to train a model that generates similar labels from new images that we convert to objects for quantitative analysis. Alternatively, an image may have a single associated label—perhaps based upon a pathologist's assessment, or other available data—and the model should also make a single prediction, such as mutational status or patient outcome.

Traditionally, machine‐learning models have been based on techniques such as random forests, support vector machines, and logistic regression [29, 30]. The algorithm developer chooses features from the image that could be relevant for prediction, and which will be provided as model inputs. These features are often the result of applying common image processing operations (e.g. convolutional filters with predefined kernels), although they might also be determined from objects previously detected in the image (e.g. the size, shape,and density of nuclei). Thus, image processing is still involved, but the developer does not explicitly define all the operations; rather, they tune the algorithm indirectly through the choice of training data, features, and model.

Deep learning refers to a subset of machine‐learning methods that have proven particularly powerful across multiple domains [1, 2]. For imaging applications, a deep‐learning model is frequently a type of convolutional neural network (CNN). As the name suggests, a CNN also relies upon convolution to generate features—however, the kernels themselves are learned during training. This has a profound impact upon what can be achieved. By learning hundreds of such convolution filters and applying them in combination with other nonlinear transforms and resizing operations, deep learning effectively frees us from the limits of human imagination in defining the input features. In practice, this makes it possible to identify far more complex or subtle patterns than could be found using other contemporary approaches—at a cost of requiring much more computational power.

### Comparison of techniques

This very brief overview aims to demonstrate the overlaps between image processing, machine‐learning, and deep‐learning approaches to digital pathology analysis. Ultimately, all are applied to the pixel values of the image. Convolution—scaling and summing neighbouring pixel values—plays a starring role in each case.

An advantage of using conventional image processing exclusively to develop an algorithm is that the methods are well‐defined and tractable. The developer tunes performance by setting key parameters, such as filter sizes and thresholds. Simplicity is a virtue: an algorithm that uses a small number of intuitive parameters is easy to apply and adapt to work on new images, whereas a complicated, hand‐tuned algorithm is likely to be brittle and overfit to one dataset. However, simplicity is also a limiting factor: the complexity of pathology data means that image processing alone is often insufficient.

A benefit of traditional machine learning is that the developer can focus on higher‐level questions: instead of devising fixed rules, they can provide training data and labels representing the images that should be handled. Developing and applying traditional machine‐learning models can also be very fast: a model can be trained in a matter of seconds while interactively annotating an image, and progressively refined if required [13], although a more structured approach to model training across multiple images is usually preferable. However, even with extensive training the success will ultimately depend upon the usefulness of the input features, which may simply not be informative enough. Since key parameters are wrapped up inside the model, they cannot be readily tuned to work on new images. When the algorithm fails, we need to train a new model.

Most of the observations regarding traditional machine learning also apply to deep learning, with two important caveats. The first is that training a deep‐learning model from scratch is usually much slower: typically requiring several hours or more, depending upon the model, training data, and computational resources available—although this can be substantially reduced if the training can instead be applied to fine‐tune an existing model. The second is that the model performance is much less limited by available features, although it remains constrained by the available training data and definition of the loss function.

**Figure 1:**
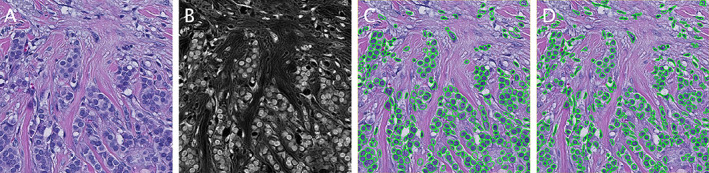
Nucleus segmentation using image processing, with and without deep learning. (A) Original H&E image. (B) Result of processing the image using colour deconvolution and image filtering to extract the haematoxylin information. (C) Segmented nuclei using a publicly available StarDist deep‐learning model trained for fluorescence data [44]. By using the processed image as input rather than the original, the model can achieve reasonable nucleus segmentation performance despite not being trained for H&E images. (D) Result of QuPath's built‐in cell detection using conventional image processing. The StarDist deep‐learning approach results in more regular contours and better handles densely‐packed regions, although the total number of nuclei detected are similar (319 and 331, respectively).

In practice, all techniques have strengths and weaknesses. Sophisticated analysis problems usually require elements of them all to be combined **(**Figure 1 **)**.

## Analysis in practice

Anyone seeking to develop or apply digital pathology methods encounters many of the same challenges. Here I discuss some of the main difficulties, and consider ways they may be addressed.

### Generalisation and bias

Limited generalisation affects all digital pathology analysis, and has been described as ‘probably the single most important obstacle for wide‐scale implementation of [computational pathology] techniques in the clinic’ [1]. A seemingly successful algorithm can be thwarted in different—and often subtle—ways when confronted by data that differs appreciably from that used for development. For example, the top‐ranked deep‐learning algorithms in the CAMEYLON17 grand challenge for detecting lymph node metastases were all reported to struggle with benign areas that occurred rarely in the training set [31]. Another study reported improved robustness by using both a much larger training dataset and weaker annotations (i.e. slide‐level labels, rather than contours outlining individual metastases), but still acknowledged a drop in performance when slides used for training and testing were acquired from different sources, or using different scanners [7]. Subtle, subvisual changes in input images can also result in quite different predictions using some deep‐learning approaches, which can even be used as a form of ‘attack,’ whereby an image is deliberately manipulated to cause a different prediction [32].

One strategy to address this is to include more diverse training images from different sources, acquired using different scanners. A problem, however, is that this can introduce learnable hidden variables, and thereby batch effects [33]. For example, a study applying deep learning to melanoma slides from five institutions demonstrated that it was possible to learn information about slide origin, scanner type, patient age, and even (to a lesser extent) slide preparation date [34]. A similar study showed that site‐specific signatures are identifiable within the images in The Cancer Genome Atlas (TCGA), and these have a relationship to ethnicity [35]. Such effects may provide an inflated estimate of accuracy or a systematic bias if the model learns characteristics that correlate with the training labels due to the cohort makeup, rather than the disease.

Another way to broaden the training data is to augment it using image‐processing operations that deliberately introduce random variations (e.g. in resolution, rotation, colour, and sharpness), thereby pushing the model towards learning more informative features [36]. Alternatively, one can take the reverse approach of making the model inputs more standardised at the prediction stage by stain normalisation [37, 38]. These methods are not exclusive, and the best results may be achieved by both broadening the model training with diverse and augmented data, and then narrowing the input variation with stain normalisation [36].

### Cell detection

The problem of generalisation is particularly clear in the continued struggle to accurately detect cells. This is a fundamental part of many pathology analysis workflows. Although few topics in bioimage analysis have received as much attention as nucleus segmentation, a review in 2012 describes how it remained unsolved after more than half a century of effort [39]. The last decade has seen substantial progress using deep learning, with hints that a single solution that handles *most* tissue, staining, and scanning variation is achievable [40, 41, 42, 43, 44]. Nevertheless, more work is needed devise accurate, robust, and computationally‐efficient cell detection methods that are incorporated into widely‐used software. This is particularly important because failures in cell segmentation are not randomly distributed, but rather tend to increase with specific morphologies.

### Boundaries and cutoffs

A benefit of image analysis is that it enables us to quantitatively answer more detailed questions from images. An inconvenience is that, freed from the imprecision of human visual estimation but lacking the expert's intuition, we are pushed to define what should be assessed to a higher degree of exactness. This means imposing hard boundaries where none may exist. For example, the area of a tumour region may be precisely determined—but only if one accepts a precise boundary defining the tumour. Pathologists annotating tumour regions draw quite different contours, each of which may be justifiable for a particular purpose, but each of which would have a different area [45]. Accepting the lack of a definitive ground truth, one may argue for the value of digital pathology because it reduces human subjectivity and improves reproducibility. However, the reality is more complicated. The problem of limited generalisation means that the algorithm *may* produce one boundary for an image scanned using a particular scanner, but quite different boundaries if the same slide is scanned on a different scanner. The extent to which this occurs needs to be explored case‐by‐case, but one should avoid assuming *a priori* that a digital method will reduce variation.

**Figure 2:**
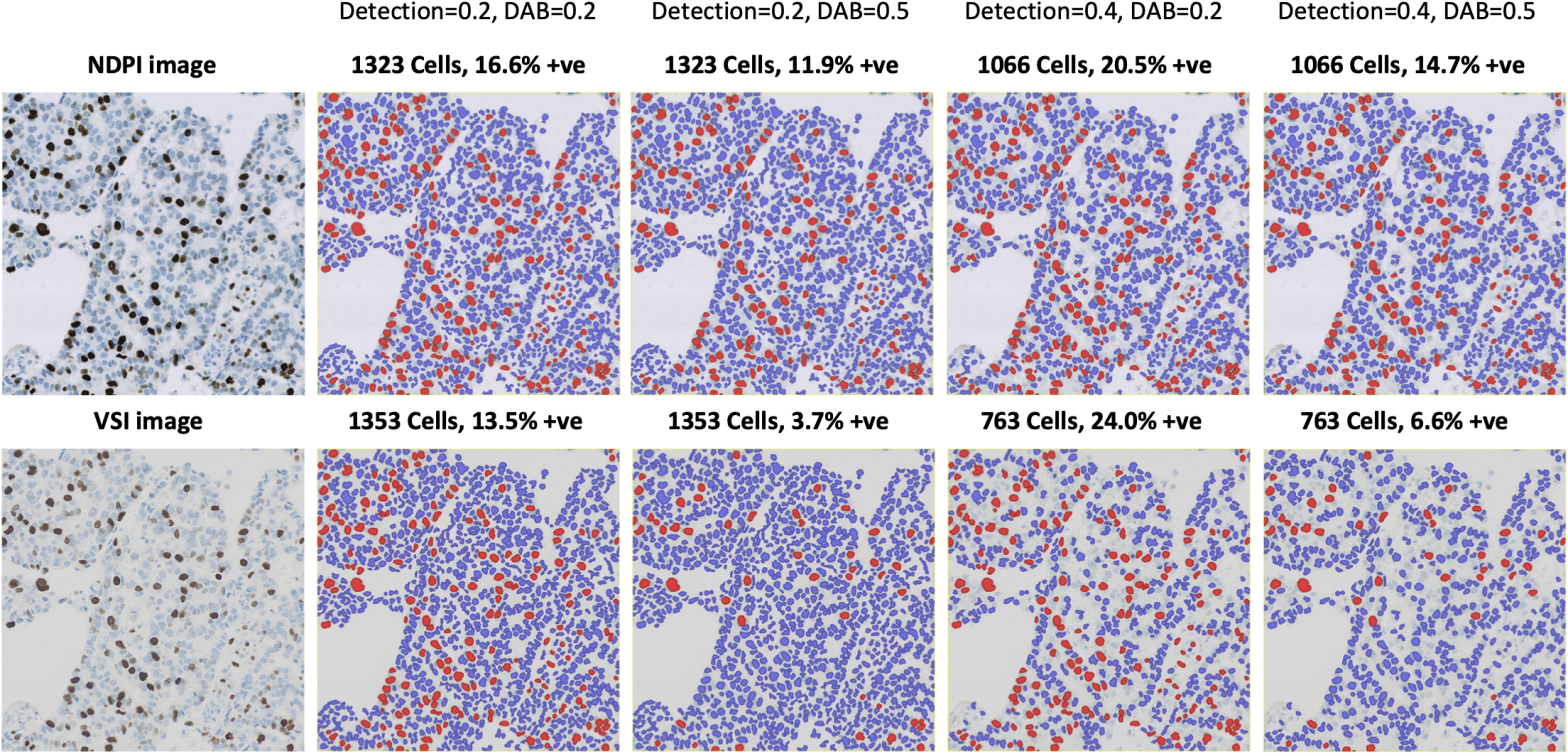
The impact of algorithm parameters and cutoff thresholds on images. QuPath's ‘positive cell detection’ command is used to determine Ki67 labelling indices for the same field of view, acquired using two different scanners. This conventional image‐processing algorithm uses multiple adjustable parameters, although here only the thresholds for nucleus detection and DAB positivity are varied. Horizontally adjacent images are from the same scanner, while vertically adjacent images are generated using the same thresholds. Detected nuclei are shown as red or blue, depending upon whether they are classified as positive or negative, respectively. Changing either threshold or the scanner can substantially change the results, although typically in predictable ways (e.g. a high detection threshold leads to negative nuclei being missed, and the labelling index is inflated; a high DAB threshold leads to positive nuclei being misclassified as negative, and the labelling index is reduced). Combining this knowledge with a careful evaluation of the markup images, it is possible for a user to identify and address many errors by adjusting algorithm parameters accordingly.

Similarly, when analysing immunohistochemistry (IHC) images, a cell may be classified as ‘positive’ or ‘negative’ (or 1+, 2+, 3+) by applying fixed cutoff thresholds based upon a summary measurement of the pixel values within the cell. Given that measured staining intensity is effectively a continuous variable, the precise choice of threshold can have a considerable impact upon output metrics—such as positive percentage, H‐score [46, 47], or Allred score [48]—if a significant proportion of cells are measured close to the threshold value. At first glance, digital scoring still seems preferable to relying upon a pathologist's visual impression, since the digital cutoff can be strictly defined and fully reproducible. However, if the threshold is held constant but the image would have different colour characteristics—perhaps due to staining variations, tissue thickness, or the choice of scanner—the analysis would still yield different results. Locking the algorithm parameters is therefore not in itself sufficient for reproducible analysis, unless one can also lock all other preanalytical variables that will impact the pixel values **(**Figure 2 **)**.

One might wish to mitigate this by avoiding hard thresholds, and using instead continuous measurements of staining intensity. While image analysis software can certainly derive numbers from such staining, these remain subject to imaging variations—and can easily be overinterpreted, e.g. if one ignores the biophysical properties of the diaminobenzidine (DAB) substrate [49]. Another tempting way to avoid the issue is to use machine learning to determine IHC positivity. This too requires caution, since it reduces tractability without necessarily improving results. For nuclear markers such as Ki67, we need to be careful that confounding features (e.g. nucleus size) cannot unduly influence any model prediction. Nevertheless, a machine‐learning approach may be justifiable for markers exhibiting complex staining patterns (e.g. PD‐L1), for which too much information is lost by simple summary metrics of staining intensity.

There are, of course, other data‐driven and adaptive approaches to determining IHC positivity cutoffs [50, 51]. The key point is that image analysis using digital pathology can give a range of plausible results based upon the interaction of the algorithm, its parameters, and the data under consideration—including all preanalytical sources of variation. We should continually resist the ‘illusion of objectivity’ by recognising potential errors and limitations, and incorporating this knowledge into how we design algorithms and interpret their outputs [52].

### Working across disciplines

These observations should not be interpreted as undermining the importance of digital pathology, nor suggesting that it cannot or does not reduce subjectivity; rather, they emphasise that it will not do so *necessarily*. Generalising is hard, there are many pitfalls, and validation details matter. Developing truly robust methods requires expertise from a range of disciplines—including pathology, histology, imaging, image processing, machine‐learning, statistics, and epidemiology—to come together. These problems are being tackled, including through increased attention being given to ‘explainable AI’ for digital pathology [53] and the creation of guidelines for the use of AI in clinical trials [54, 55]. Software and algorithm developers can help by documenting their design decisions, drawing attention to known weak points and key parameters, and providing visualisations that make it easier for others to understand what the analysis is really doing.

Detection and classification remain the most difficult (and error‐prone) steps for most quantitative analyses. False‐positives and ‐negatives occur even when using a deep‐learning approach—particularly in the presence of mimics or artefacts. On the other hand, counting and quantification are often trivial from a computational perspective. This largely represents an inversion of a human pathologist's skillset: an experienced pathologist may reliably distinguish the structures of interest while ignoring mimics and artefacts, but cannot feasibly count a million classified cells or precisely determine a 1‐mm^2^ hotspot by eye alone. This suggests that an optimal arrangement may combine the strengths of human and computer, and factor the limitations of algorithms into how they are used. One pragmatic solution for quantitative analysis is to require manual input when defining a region of interest, to steer the analysis away from challenging areas or artefacts. A proposed approach for AI‐based assessment is to use AI as a form of triage, prioritising sensitivity over specificity [7].

## Implementation, accessibility, and openness

The difficulty of developing robust, fully‐automated analysis methods may partly explain why few digital pathology algorithms have moved beyond publications into becoming available to pathologists. However, algorithmic challenges are only one consideration in the creation of useful digital pathology tools.

### Proof‐of‐concept versus usable methods

Research groups developing computational methods have traditionally published articles describing their own bespoke algorithms, validated on their own in‐house datasets. If code and data are shared at all, it is often ‘by reasonable request’. This presents a considerable barrier to anyone who might wish to test whether the method works on their own data: they need permission and support from the original authors, who may be unable or unwilling to provide it [56]. The alternative is to try to reimplement the method from the published description—but this inevitably involves considerable effort, and guesswork where the description is incomplete [56].

This closed, ‘share‐on‐demand‐(perhaps)’ approach assumes that the key research contribution of a new computational method is the idea described in the article—relegating implementation to a technical detail [57]. However, implementation within software is crucial for the method to be used by anyone, and real‐world use determines the true value of the idea [39, 58].

The situation is improving as open research becomes required by some funders and journals [59, 60]. ‘Open’ does, however, afford considerable room for interpretation [61]. Although more papers include code as supplementary material, what is publicly shared is seldom sufficient to replicate the analysis or apply the method to new data [56]. The data used to train an AI model and the AI model itself are rarely made available—meaning that any researcher wishing to test a published method must still replicate a large amount of the work in the original study to train their own model. This means it can take weeks or months of effort to fill in the gaps to find out whether an ostensibly ‘open’ method works or not. There is often little incentive for someone with the requisite knowledge to put in the effort, because it is time not spent developing the ‘new’ methods they may need to advance their own careers. This contributes to a scientific literature replete with novel algorithms and proofs of concept, but few usable implementations available to the wider community. Different groups reinvent similar techniques, and there is a substantial disconnect between what seems possible (based on the literature) and what is possible (based upon available software).

### Complications of sharing

The reasons for this are understandable: sharing data and code is hard. On one side, whole‐slide images are large: making them available requires data storage and bandwidth, which come at a cost. The images are also typically saved in bespoke, scanner‐specific file formats that include additional labels and metadata; this makes them difficult to anonymise both fully and with confidence that nothing has been missed [62]. Sharing also raises important questions around how the images may be used, including whether it is permitted to develop commercial AI models based on them; these must be resolved, with clear license statements provided, for others to safely use the shared data [63, 64]. While each may be surmountable, this combination of technical, financial, legal, and ethical hurdles can make sharing raw images extremely difficult.

Sharing code is also not without risk and complication [65, 66, 67]. An obvious risk is that the code may simply not do what it should. Bugs are inevitable in any sophisticated software, and researchers who share their code expose themselves to criticism—even retraction—if their software is shown not to do what is described in the paper [68]. But even if it works as described, making software open involves taking on a lot of additional work and responsibility [69, 70]. Code intended for public consumption typically needs to be of a higher standard than in‐house code; one cannot make simplifying assumptions (e.g. only supporting image files from a single scanner), and must pay considerably more attention to code‐quality, portability and ease‐of‐installation. Appropriate software licenses should be chosen [71], which can involve securing agreement from a range of stakeholders with different priorities (including principal investigators, funders, innovation departments). Then, if the software has sufficient appeal and the authors desire to maximise its usefulness, the work is really only beginning: users require documentation and ongoing support, perhaps lasting far beyond the grant that originally funded the work [69].

### Common datasets

The problem of sharing the data used to develop an algorithm can be partially circumvented by using common public datasets. Notably, the pathology slides from TCGA and The Cancer Imaging Archive (TCIA) have been widely used by the digital pathology community [15, 16, 23, 33, 72, 73, 74, 75]. This has limits: TCGA/TCIA images are not intended to be definitive and cannot be fully representative [75]—for example, the majority are of frozen sections in Aperio's SVS file format—and exclusive use of TCGA to train and validate algorithms can lead to overly‐optimistic performance assessments [35]. Nevertheless, TCGA/TCIA have proven valuable and will continue to play an important role in advancing the field, alongside new image repositories such as that being created through the IMI‐BIGPICTURE project [76].

### Grand challenges

Irrespective of whether the data are public or not, whenever a research group develops, validates, and publishes their own algorithm, it is impossible to guard against the limitations of publication bias and multiple hypothesis testing—however inadvertent this may be—because the algorithm that is published will inevitably be the one that ‘worked’. Grand challenges provide a way to address this [4]. A grand challenge involves organisers releasing a labelled dataset, along with detailed information about assessment metrics. Groups then compete to develop algorithms using these data and metrics. Crucially, the organisers withhold additional labelled data used to rank algorithm performance. Grand challenges are often held in conjunction with conferences, where the best‐performing algorithm is unveiled and the methods described in a later publication. Prominent examples for pathology include the CAMELYON16 and CAMELYON17 challenges to detect lymph node metastasis [8, 31], the GlaS challenge for colon gland segmentation [77], the BACH challenge to classify diagnostically relevant regions in breast cancer biopsies [78], and the PANDA challenge for Gleason grading of prostate cancer [25].

Grand challenges have proven extremely effective in galvanising the community—across both academia and industry—to work on solving a specific problem, with a consistent method of comparing performance. There are inevitably limitations, and impressive results in a challenge do not indicate that a problem has been ‘solved’ [31]. For example, the rankings do not necessarily reflect the algorithm that is ‘best’ in terms of generalisation and performance on most real‐world data, but rather only how algorithms fared using the specific test dataset and defined metrics [79]. Particularly when the same group may submit multiple algorithms, one can attempt to game the system or achieve a high score without necessarily developing a practical method that others may use. Nevertheless, grand challenges are open by design, and multiple groups competing in the challenge will be able to review and critique the data and metrics; this represents a major improvement over groups publishing incomparable work using their own in‐house data and assessment criteria. Recently, guidelines have been published that aim to improve the transparent reporting of challenges [80].

### Open data and software

Much modern science depends upon *Free and Open‐Source Software (FOSS)*, often referred to simply as ‘open software’. Users of FOSS tend to focus on the ‘free’ aspect in terms of ‘no financial cost’. However, proponents of open software use ‘free’ in a broader sense, referring also to the freedom to use the software for any purpose, to examine and modify the source code, and to redistribute both original and modified copies [71]. These elements are central to open software's unique contribution to research, enabling methods to not only be accessible to others, but to be interrogated, extended, enhanced, and shared by the wider community. Indeed, scanner vendors often provide image viewers that can be freely downloaded, but for which source code is not available, and numerous research publications provide software code only with restrictions (e.g. noncommercial use only); despite the lack of financial cost for the user, neither is FOSS.

Sharing digital pathology tools under a recognised FOSS license goes some way towards enabling them to be used freely by others. However, ‘software’ itself is a broad term: much software exists in the form of code libraries or scripts that lack the user‐friendly interface and visualisation capabilities required by nonprogrammers. Components such as these must be incorporated into larger software applications to be directly useful for pathologists. This larger application may or may not then be open itself; many companies integrate open‐source components into their proprietary software.

Developing, documenting, and supporting comprehensive software applications is complex, challenging, and time‐consuming [69, 81]. It is infeasible—and undesirable—for individual research groups to develop their own complete software applications simply to make their algorithms more usable. Fortunately, it is also not necessary. Several open software platforms exist that can solve the majority of the challenges around data management, visualisation, and interactivity while permitting others to add new algorithms and functionality for specific tasks.

The most important demonstration of this in the bioimaging community is ImageJ (NIH, Bethesda, MD, USA). Over more than a quarter of a century, ImageJ has dominated bioimage analysis and a recent review listed its direct precursor, NIH Image, as one of the ‘ten computer codes that transformed science’ [82]. Extensibility is key to ImageJ's continued success: when developers release new algorithms as ImageJ macros or plugins, users can install and run them, without needing to learn a whole new interface [83]. Several other bioimage analysis applications with a similar philosophy were later developed, including 3D Slicer [84], CellProfiler [85], and Icy [86].

All of these predate the growth of digital pathology, and none were designed to handle the specific challenges of whole‐slide image analysis. A few ImageJ plugins for pathology applications enable relatively straightforward processing techniques to low‐resolution images or cropped fields of view [87, 88]. Without full whole‐slide or AI support, these have limited use nowadays, but were nevertheless important in demonstrating the value of open methods being available for pathologists. More recently, several new open‐software applications have been developed with whole‐slide image analysis as a primary goal [89], including TMarker [90], Orbit [91], and QuPath [13] (desktop applications), and Cytomine [92] and the Digital Slide Archive [93] (web‐based platforms).

The importance of making research software that is open and flexible extends beyond enabling the analysis from a single paper to be reproduced. To take one example, QuPath's original publications had a narrow focus on colon and breast cancer biomarkers [13, 94], but the software has since been further developed and used in over 1,000 published journal articles across a wide range of diseases and applications. Openness means that the strengths and limitations of the tool can be freely explored, independently of the author's claims, and numerous studies have compared QuPath‐based analysis with both other software and with manual evaluation [95, 96, 97, 98, 99]. Groups using QuPath have made their scripts, extensions, and protocols available to aid reproducibility, in a way that could not be achieved without open software [10, 100, 101].

The value of openness applies not only to code and data. The Scientific Community Image Forum (https://image.sc) was established to help improve the practice of scientific image analysis [102]. It is now the primary discussion channel for more than 40 open‐software projects, including ImageJ, QuPath, Orbit, and Cytomine [69]. The forum aims to be inclusive and collaborative, with users from many disciplines contributing to discussions. Currently, image.sc contains over 200,000 posts across more than 28,000 different topics—each tagged and searchable—with more added each day. The forum acts both as a substitute for paid software support and as a venue for cross‐disciplinary discussions, while simultaneously providing community feedback directly to developers.

## Outlook

The potential of deep learning for AI‐based assessment of clinical samples has been firmly established. Nevertheless, significant challenges remain concerning generalisation and validation if we are to see robust algorithms come into widespread clinical use [1]. These challenges cannot be adequately addressed by individual groups working solely with in‐house or public data. Rather, they will require long‐term projects incorporating insights from multiple disciplines, large and diverse datasets, and innovative approaches to training AI models at scale [62].

In the meantime, digital pathology analysis is already widely used. This is driven by the proliferation of whole‐slide scanners in research institutes, which has dramatically increased the number of scientists using histological samples for basic biology, preclinical, and veterinary studies. These data include H&E alongside other stains and IHC markers, across tissues, species, and image types—including multiplexed immunofluorescence and imaging mass cytometry data, the value of which can only be realised through computational analysis [103, 104]. The ubiquity brings concerns: digital pathology in no way changes the fact that biologists should engage with pathologists to properly evaluate such data [105, 106], but rather adds a need to understand the computational methods used as well. This is made difficult by a lack of pathologists, image analysis specialists, and capable software tools to meet the growing and varied demand [106, 107].

The growth of open science enables anyone with an interest in the field to contribute directly. This includes sharing data and code under open licenses where possible. Developers know that custom algorithms and AI‐models will often fail on new data, but this is no reason to withhold them: the limitations of the field need to be understood if they are to be addressed. In some cases, providing open software will enable more researchers to analyse their own data efficiently and effectively; in others, it may lead to finding that the software does not work as expected. Either outcome provides more value to the scientific community than a published method that lacks any usable implementation.

In the past, a pathologist engaging in digital pathology algorithm development might expect to spend hours laboriously outlining thousands of image regions, with no clear path leading to software they could later use. This should no longer be the case. Devising efficient annotation strategies is a computational problem that can be solved, while developers can make their algorithms accessible through open‐software platforms if they wish. Through initiatives like the Scientific Community Image Forum, pathologists can directly engage with algorithm and software developers, sharing their expertise and expressing their needs [102]. By understanding the strengths, challenges, and incentives of researchers and companies who develop digital pathology software, we can all contribute to a scientific culture that enables the field to advance more rapidly.

## Author contributions statement

PB wrote the manuscript.

## Data Availability

A short overview of the image processing terms used in this review is available at https://petebankhead.github.io/2022-image-processing-overview. The images and scripts used in the preparation of figures for this article are available at https://github.com/petebankhead/2022-qupath-cell-detection.
